# The “Porto Alegre HIV Charter”: Confronting the HIV epidemic in Rio Grande do Sul, Southern Brazil

**DOI:** 10.1016/j.bjid.2026.105965

**Published:** 2026-09-09

**Authors:** Tarsila Vieceli, Cezar Vinícius Würdig Riche, Viviane Raquel Buffon, Cristiane Pimentel Hernandes, Diego R. Falci, Gerson Junqueira, Maria Leticia Ikeda, Mauricio Roth Volkweis, Ricardo S. Diaz, Alessandro C. Pasqualotto, Dimas A. Kliemann

**Affiliations:** a Universidade Federal de Ciências da Saúde de Porto Alegre (UFCSPA), Porto Alegre, RS, Brazil; b Santa Casa de Porto Alegre, Porto Alegre, RS, Brazil; c Faculdade de Medicina, Universidade Luterana do Brasil, Canoas, RS, Brazil; d Faculdade de Medicina da Universidade de Caxias do Sul, Caxias do Sul, RS, Brazil; e Faculdade de Medicina da Universidade de Santa Cruz do Sul, Santa Cruz do Sul, RS, Brazil; f Faculdade de Medicina, Pontifícia Universidade Católica do Rio Grande do Sul, Porto Alegre, RS, Brazil; g Infectious Diseases Service, Hospital de Clínicas de Porto Alegre, Porto Alegre, RS, Brazil; h Associação Médica do Rio Grande do Sul (AMRIGS), Porto Alegre, RS, Brazil; i Universidade do Vale do Rio dos Sinos, São Leopoldo, RS, Brazil; j Oral Diseases Team, Conceição Hospital Group and Brazilian Scouts Association, Porto Alegre, RS, Brazil; k Escola Paulista de Medicina, Universidade Federal de São Paulo, São Paulo, SP, Brazil; l University of Minnesota, Minneapolis, MN, USA; m Grupo Hospitalar Conceição, Porto Alegre, RS, Brazil

Brazil has long been recognized for one of the most ambitious public responses to HIV in the world, anchored in universal, cost-free access to antiretroviral therapy through the Unified Health System (SUS) since the 1990s. That investment continues to yield results: in 2024 the country recorded the lowest number of AIDS-related deaths in three decades, and national AIDS mortality fell by roughly a third over the previous ten years.^1^ Yet HIV remains a major public health problem and, crucially, its burden is not evenly distributed. Nowhere is this paradox sharper than in Rio Grande do Sul (RS), the southernmost state with a strong, decentralized health network and a tradition of social organization, which nonetheless leads the country in AIDS mortality.

This concentration of disease is, on its face, a conundrum. Rio Grande do Sul is one of the most developed of Brazil’s federative units: home to roughly 11 million people, it is the country’s sixth most populous state and among its most urbanised, and its social indicators sit near the top of the national ranking ‒ a Human Development Index in the ‘very high’ band (approximately 0.81, among the highest of the 27 federative units), an adult literacy rate close to 97% (well above the national average), and the longest life expectancy in the country.^2^^,^^3^ HIV epidemics of this magnitude are usually expected to track poverty, low schooling, and fragile infrastructure; in RS, they do not. That a comparatively wealthy, highly literate, and well-served state should nonetheless lead Brazil in AIDS mortality forces a different reading of the problem: one centred not on underdevelopment, but on who reaches care and how quickly, and without stigma.

The numbers are difficult to overstate. RS accounts for a share of the national HIV/AIDS burden well above its population weight: it is among the states that have historically reported the largest absolute numbers of AIDS cases since the beginning of the epidemic, and its AIDS detection rate ‒ around 24 cases per 100,000 inhabitants in recent years - sits persistently among the highest in Brazil^4^ (Fig. 1). Most strikingly, RS has repeatedly ranked first among all Brazilian states in AIDS mortality.^1^^,^^4^ Recent epidemiological data further underscore the magnitude of this crisis: in the metropolitan region of Porto Alegre, approximately one in every 45 young adults aged 30‒45 years is currently living with HIV, revealing an alarmingly high prevalence in a population that should be at the center of prevention efforts.^5^ The epidemic is concentrated in, but not confined to, the Porto Alegre metropolitan region and the densely urbanized corridor that runs north toward the Serra and the Sinos valley.

**Fig. 1:**
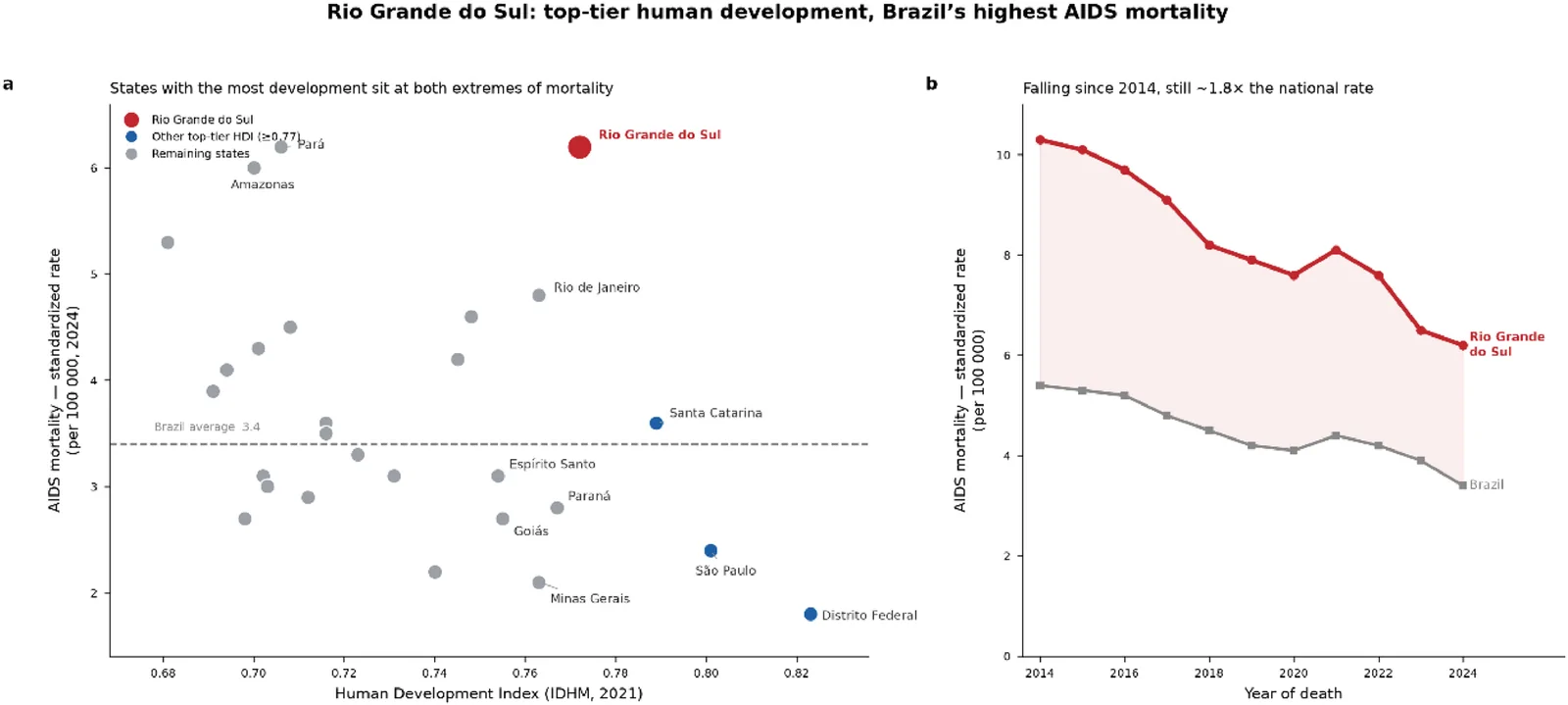
Rio Grande do Sul: Top-tier human development, Brazil’s highest AIDS mortality. (a) States with the most development sit at both extremes of mortality. (b) Falling 2014, still ∼1.8 × the national rate.

Beyond issues of access and late clinical presentation, the unique genomic landscape of the virus in Rio Grande do Sul presents a formidable biological hurdle. Unlike the rest of Brazil, where other subtypes prevail, the regional epidemic is driven primarily by HIV-1 subtype C and the CRF31_BC recombinant form.^6^ These specific viral lineages possess a Reproductive number (R0) estimated to be significantly higher than other strains endemic to the country.^7^ Notably, subtype C exhibits a distinct competitive advantage in *in vitro* heterosexual transmission models,^8^ a characteristic that has allowed it to displace other clades across much of the African continent.^9^

Porto Alegre itself stands at the centre of this picture. In composite analyses combining general-population detection, mortality, and detection in children under five, the state capital has emerged as the most affected capital in the country, and it carries the highest AIDS mortality coefficient among Brazilian capitals.^1^^,^^10^ The phenomenon is regional rather than isolated: six RS municipalities appear among the 100 most affected Brazilian cities with more than 100,000 inhabitants, including Canoas, Gravataí, Novo Hamburgo, Bagé, Pelotas, and Passo Fundo; the former three are part of the Porto Alegre metropolitan region. An epidemic of this magnitude, in a state of this capacity, is not a problem of resources alone: it is a problem of access, of timing, and of stigma.^11^

There is, however, clear evidence that coordinated action changes the trajectory. The single most encouraging signal in RS comes from the prevention of vertical (mother-to-child) transmission: AIDS detection in children under five fell by more than 70% between 2013 and 2023.^5^ This decline did not happen by chance. It followed structured investment: priority municipalities, regionalized care and dedicated mortality committees, expanded maternal and partner testing, and a state effort toward certification of the elimination of vertical transmission. The lesson is unambiguous: when the response is organized, decentralized, and adequately funded, the curve bends. The question that this Alliance seeks to answer is why the same logic has not yet been applied, with equal force, to the adult epidemic. Part of the answer lies in the HIV care cascade. Nationally, an estimated 9 in 10 people living with HIV are aware of their diagnosis, but important regional disparities persist. In Rio Grande do Sul, recent population-based serological data indicate that only 63% of people living with HIV were previously aware of their diagnosis, revealing a substantial diagnostic gap.^5^ A smaller proportion are retained on antiretroviral therapy; among those treated, viral suppression is excellent (around 95%).^1^^,^^12^ The principal gaps, therefore, are not in the efficacy of treatment but in timely diagnosis and durable linkage to care. Late diagnosis remains a central driver of both mortality and onward transmission, and it falls hardest on those already made vulnerable by stigma, poverty, and discrimination: young Men who have Sex with Men (MSM), transgender people, sex workers, people who use drugs, and people living on the urban periphery. Stigma is not a peripheral concern here; it is a clinical and epidemiological force that delays testing, fractures treatment, and costs lives.

What makes the present moment different is that science has placed in our hands tools capable of changing the course of this epidemic. The first is diversified HIV testing. Conventional laboratory testing, point-of-care rapid testing, and HIV self-testing together allow diagnosis to move out of the clinic and into the community, reaching people earlier and on their own terms. Decentralizing the diagnostic offer (into primary care, pharmacies, mobile units, and trusted community spaces) is the single most powerful way to close the first gap in the cascade.^13^

The second is combination prevention, which has been transformed by long-acting Pre-Exposure Prophylaxis (PrEP). Oral PrEP (tenofovir/emtricitabine) has been available through SUS since 2017.^14^^,^^15^ but daily adherence is difficult precisely for those at highest risk. Long-acting injectable Cabotegravir (CAB-LA), administered every two months, addresses that gap directly. In the pivotal HPTN 083 and HPTN 084 trials, CAB-LA was superior to oral PrEP in preventing HIV,^16^^,^^17^ and the World Health Organization recommended it as part of standard prevention in 2022.^18^ CAB-LA was approved by Anvisa in 2023 and became available in the Brazilian private market in August 2025 ‒ at roughly R$ 4000 (USD 780) per dose, more than three times the Brazilian minimum wage. Brazilian implementation data are compelling: in the Fiocruz-led ImPrEP CAB-Brasil study, conducted across six capitals, the great majority of participants preferred the injectable, on-time attendance exceeded 90%, and no participant on cabotegravir acquired HIV.^19^

This is where equity becomes decisive. A prevention tool that exists only in the private sector will not reduce a public epidemic; worse, it risks widening the very inequalities that drive HIV, delivering the benefits of adherence to those least exposed while leaving the most vulnerable dependent on a method they struggle to sustain. The incorporation of long-acting PrEP into SUS ‒ now under evaluation by Conitec, the Brazilian regulatory agency ‒ must therefore be understood not as a luxury but as a public-health priority. Access must be broad, dignified, and decentralized.

The third tool is treatment itself. Immediate, ideally same-day, initiation of antiretroviral therapy for all people diagnosed with HIV (excluding those who require relay-ART start) improves outcomes and shortens the window of transmission. The evidence underpinning Undetectable = Untransmittable (U = U; I = I, *Indetectável = Intransmissíve*l) is now beyond serious dispute: people on suppressive therapy with an undetectable viral load do not transmit HIV sexually, as demonstrated by the PARTNER and HPTN 052 studies^20^^,^^21^ and formally endorsed in Brazil’s national messaging. Therefore, treatment is prevention. Closing the linkage-and-retention gap in RS is, simultaneously, the most humane and the most effective prevention strategy available to us. In that sense, newer therapeutic options, such as bictegravir / emtricitabine / tenofovir alafenamide (a triple therapy that is safe for patients with kidney failure), and new injectable antiretrovirals, such as Cabotegravir plus Rilpivirine, may help closing this gap. As demonstrated in the LATITUDE trial, monthly injections of long-acting ART can be a promising strategy in patients with adherence challenges.^22^

It is against this background that, on 23 May 2026, a broad coalition gathered in Porto Alegre ‒ physicians and other health professionals, activists, public managers, medical and scientific societies, civil-society organizations, and people living with HIV ‒ and constituted itself as the *Aliança Gaúcha pelo Enfrentamento do HIV (Gaúcha Alliance to Confront HIV)*. In the same spirit that produced earlier Porto Alegre and Manaus declarations on neglected and HIV-associated diseases,^23^^,^^24^ the Alliance issued the Porto Alegre Charter, a collective commitment of the gaúcho people to confront HIV with science, compassion, and unity. Its core commitments are summarized in Table 1, and the full text is reproduced as an Appendix.

**Table 1:** Commitments of the Porto Alegre Letter.

Two principles frame these commitments: people on suppressive treatment with an undetectable viral load do not transmit HIV (U = U), and ending the epidemic depends on access, information, and continuous care.
• Disseminate quality information and combat HIV-related stigma.
• Expand universal testing and early diagnosis across the entire state.
Guarantee broad, dignified, and decentralized access to oral and injectable PrEP.
• Strengthen immediate and continuous treatment for all people living with HIV.
• Promote humanized care, free from discrimination and prejudice.
• Integrate civil society, health services, universities, medical societies, and public managers in a collective response.
• Listen to and welcome people living with HIV, recognizing their dignity, their voice, and their rights.

Bending the mortality curve will require confronting why people still die and still disengage from care. The systematic review of HIV/AIDS deaths ‒ through dedicated mortality (death-review) committees, of the kind already established for maternal and infant mortality in Brazil ‒ would allow each death to be examined for missed opportunities along the cascade, turning fatal outcomes into corrective action.^1^ Although there is a Committee on Mortality related to HIV/AIDS in Porto Alegre, the generation of death-review reports would be a useful tool to guide preventive strategies. Studies evaluating mortality in PLHIV in RS demonstrate that factors associated with mortality are not always biomedical.^25^ They include low health literacy in the general population; fragile systems for treatment adherence and retention, which call for active linkage, the tracing of people who miss appointments, and flexible service hours rather than passive scheduling; and the heavy, frequently unaddressed burden of mental illness and substance use among people living with HIV, which must be met with integrated psychosocial and harm-reduction support rather than exclusion from care. Above all, primary care must be equipped and trained to deliver HIV testing, treatment initiation, and continuity, decentralising the response to where people actually live and freeing specialised services to manage clinical complexity. In one study evaluating predictors of mortality of PLHIV in Porto Alegre, the factor most strongly associated with mortality was being ART-naive, and a third of the patients were recently diagnosed, highlighting a major proportion of patients diagnosing HIV at late stages of disease. This demonstrates that the public system is failing at diagnosing and treating patients at an early stage, where complications such as severe opportunistic infections are usually avoided.^25^

Additionally, structured care focused on symptomatic patients with advanced AIDS have a great impact on reducing mortality. This is not a theoretical proposition: a structured, specialist-led inpatient model of care for advanced HIV disease was recently evaluated in a before-and-after study of 963 hospitalizations among 899 patients in Porto Alegre, comparing pre-and post-implementation period. The model substantially intensified diagnostic practice: urine TB-LAM testing rose from 38% to 88% of admissions, cryptococcal antigen from 40% to 83%, Histoplasma antigen from 42% to 62%, and lumbar puncture from 20% to 36%, while microbiologically confirmed tuberculosis increased (26% to 41%) as empiric TB diagnoses fell (21% to 10%). Inpatient ART initiation rose from 24% to 72%, and the median time to ART shortened from 10 to 6 days. These process gains translated into better outcomes: in-hospital mortality fell from 24% to 17%, ICU admission from 31% to 21%, and the composite of ICU admission or death from 44% to 33%. After adjustment, hospitalization in the post-implementation period was independently associated with a 26% lower risk of in-hospital death (adjusted risk ratio 0.74; 95% CI 0.57–0.96), a 30% lower risk of ICU admission (aRR = 0.70; 0.57–0.86), and a 22% lower risk of the composite adverse outcome (aRR = 0.78; 0.67–0.92). Organizing inpatient care around the advanced-HIV-disease package, in short, does more than improve diagnosis: it keeps people alive.^26^

Even where diagnosis and treatment is ultimately reached, the cost of late presentation is still being paid in lives. A substantial share of HIV-related deaths in RS occurs among people who arrive with advanced HIV disease, in whom opportunistic infections ‒ tuberculosis, cryptococcal meningitis, disseminated histoplasmosis, pneumocystosis, and others ‒ remain leading and largely preventable causes of death.^25^ Closing this gap depends on diagnosis as much as on drugs. A defined package of rapid, point-of-care tests should be consistently available wherever advanced disease is managed: serum and cerebrospinal-fluid cryptococcal antigen, urinary Histoplasma antigen, urine lipoarabinomannan (TB-LAM), and rapid molecular tuberculosis testing (for example, Xpert MTB/RIF) where laboratory capacity allows.^23^^,^^27^, ^28^, ^29^ A study where evaluating the introduction of point-of-care diagnostics for tuberculosis, histoplasmosis and cryptococcosis in routine HIV care in several hospitals, most in RS, found that POC testing increased for TB, (1.8-fold) cryptococcosis (2.8-fold), and histoplasmosis (2.8-fold) compared to historical data.^30^ Equally important is clinical suspicion: histoplasmosis in particular remains underdiagnosed in southern Brazil, and only a clinician who considers it will test for it. Embedding advanced-HIV-disease screening packages, together with the training to use them, into the routine care of every person presenting with a low CD4 count is among the most immediate ways to lower HIV mortality in our state.

Finally, no response to HIV in Rio Grande do Sul can succeed while it continues to treat mental illness and substance use as reasons to withhold care rather than as treatable comorbidities that demand it. Psychiatric conditions and alcohol and other drug use - including the crack cocaine use that marks parts of our urban epidemic - are among the strongest determinants of late diagnosis, interrupted linkage, non-adherence, and loss to follow-up, and they fall most heavily on the same populations already burdened by stigma and poverty.^31^ Labeling these patients as “non-adherent” and discharging them to fail is both a clinical and an ethical error. What is needed instead is structured, integrated care: routine screening for psychiatric and substance use disorders at every point of entry; mental health and addiction services embedded within HIV care rather than merely referred from it, in articulation with Brazil’s psychosocial care network (CAPS and CAPS-AD); harm-reduction approaches that meet people where they are instead of conditioning treatment on abstinence; trauma-informed, low-barrier, peer-supported models; and attention to the housing instability, incarceration, and social rupture that so often surround them. For many of these patients, long-acting injectable antiretrovirals may be the difference between repeated viral rebound and durable suppression.^22^ Stabilizing mental health and substance use is not ancillary to HIV care in our state; for a substantial share of those who are dying, it is the precondition for everything else.

Rio Grande do Sul has everything needed to bend this curve: a robust public health system, a scientific and clinical community of international standing, an organized civil society, and (in its own success against vertical transmission) living proof that determined action works. What remains is the political will to test universally, to guarantee equitable access to oral and long-acting PrEP through SUS, to treat immediately and continuously, and to dismantle the stigma that still surrounds this diagnosis. No person should be defined by a diagnosis, and in this epidemic, as in this state, no one can be left behind.

## Funding

The authors received no specific funding for this work.

## Conflicts of interest

The authors declare no conflicts of interest.
