# Supplementary material for: The “Porto Alegre HIV Charter”: Confronting the HIV epidemic in Rio Grande do Sul, Southern Brazil
Source: Braz J Infect Dis. 2026 Sep 9;30(5):105965. doi: 10.1016/j.bjid.2026.105965 (PMC13579573; doi:10.1016/j.bjid.2026.105965)
Supplement: Supplementary file 1 [file mmc1.docx]

BJID-D-26-00170 _Supplementary Material

**Appendix** The Porto Alegre Letter (full text).

**Gaúcho Alliance to Confront HIV**

*Porto Alegre, 23 May 2026*

We ‒ gaúchos and gaúchas; health professionals, activists, public managers, medical societies, social organizations, and citizens committed to life ‒ unite to declare that we will no longer accept that HIV continues to steal futures, widen inequalities, and silence people in Rio Grande do Sul.

We live in a state marked by solidarity, courage, and the capacity to rebuild. It is in that same spirit that we choose to confront an epidemic that still wounds thousands of families, especially those made most vulnerable by prejudice, misinformation, and barriers to care. We know that no one wins this fight alone. HIV cannot be confronted with fear, judgment, or indifference. It must be confronted with science, compassion, unity, and humanity.

Today, science offers us tools capable of changing the course of this epidemic. We are able to expand testing to all people, ensuring early diagnosis and rapid access to treatment. We are able to expand combination prevention ‒ including condoms, health education, oral PrEP, and long-acting injectable PrEP. We know that people in treatment with an undetectable viral load do not transmit HIV. We know that saving lives depends on access, information, and continuous care.

For these reasons, together we assume a set of fundamental commitments:

• To disseminate quality information and combat the stigma associated with HIV;

• To expand universal testing and early diagnosis throughout the state;

• To guarantee broad, dignified, and decentralized access to oral and injectable PrEP;

• To strengthen immediate and continuous treatment for all people living with HIV;

• To promote humanized care, free from discrimination and prejudice;

• To integrate civil society, health services, universities, medical societies, and public managers in a collective response;

• To listen to and welcome people living with HIV, recognizing their dignity, their voice, and their rights.

We believe that no person should be defined by a diagnosis. Every life matters. Every person deserves care, respect, and the opportunity to live fully. The fight against HIV does not belong to medicine or to governments alone ‒ it belongs to all of us.

The Porto Alegre Letter is born as a call to action and to hope: a collective commitment of the gaúcho people to life, to science, and to the future. Because only united can we transform the reality of HIV in Rio Grande do Sul.

**And because no one can be left behind.**

Translated from the original Portuguese (“*Carta de Porto Alegre — Aliança Gaúcha pelo Enfrentamento do HIV*”).

**Carta de Porto Alegre**

**Aliança Gaúcha pelo Enfrentamento do HIV**

*Porto Alegre, 23 de maio de 2026*

Nós, gaúchos e gaúchas, profissionais da saúde, ativistas, gestores públicos, entidades médicas, organizações sociais e cidadãos comprometidos com a vida, nos unimos para dizer que não aceitaremos mais que o HIV siga roubando futuros, ampliando desigualdades e silenciando pessoas no Rio Grande do Sul.

Vivemos em um estado marcado pela solidariedade, pela coragem e pela capacidade de reconstrução. É com esse mesmo espírito que escolhemos enfrentar uma epidemia que ainda machuca milhares de famílias, especialmente aquelas mais vulnerabilizadas pelo preconceito, pela desinformação e pela dificuldade de acesso ao cuidado. Sabemos que ninguém vence essa luta sozinho. O HIV não pode ser enfrentado com medo, julgamento ou indiferença. Ele deve ser enfrentado com ciência, acolhimento, união e humanidade.

Hoje, a ciência nos oferece ferramentas capazes de mudar o rumo dessa epidemia. Temos condições de ampliar a testagem para todas as pessoas, garantindo diagnóstico precoce e acesso rápido ao tratamento. Temos a possibilidade de expandir a prevenção combinada, incluindo preservativos, educação em saúde, PrEP oral e PrEP injetável de longa duração. Sabemos que pessoas em tratamento e com carga viral indetectável não transmitem o HIV. Sabemos que salvar vidas depende de acesso, informação e cuidado contínuo.

Por isso, assumimos juntos alguns compromissos fundamentais:

• Levar informação de qualidade e combater o estigma relacionado ao HIV

• Ampliar a testagem universal e o diagnóstico precoce em todo o estado

• Garantir acesso amplo, digno e descentralizado à PrEP oral e injetável

• Fortalecer o tratamento imediato e contínuo para todas as pessoas vivendo com HIV

• Promover cuidado humanizado, livre de discriminação e preconceito

• Integrar sociedade civil, serviços de saúde, universidades, entidades médicas e gestores públicos em uma resposta coletiva

• Escutar e acolher as pessoas vivendo com HIV, reconhecendo sua dignidade, sua voz e seus direitos

Acreditamos que nenhuma pessoa deve ser definida por um diagnóstico. Toda vida importa. Toda pessoa merece cuidado, respeito e oportunidade de viver plenamente. A luta contra o HIV não pertence apenas à medicina ou aos governos — ela pertence a todos nós.

A Carta de Porto Alegre nasce como um chamado à ação e à esperança. Um compromisso coletivo do povo gaúcho com a vida, com a ciência e com o futuro. Porque somente unidos conseguiremos transformar a realidade do HIV no Rio Grande do Sul.

E porque ninguém pode ficar para trás.
